# Sustainable Chitosan-Dialdehyde Cellulose Nanocrystal Film

**DOI:** 10.3390/ma14195851

**Published:** 2021-10-06

**Authors:** Cong Gao, Shuo Wang, Baojie Liu, Shuangquan Yao, Yi Dai, Long Zhou, Chengrong Qin, Pedram Fatehi

**Affiliations:** 1 Department of Light Industrial and Food Engineering, Guangxi University, Nanning 530004, China; cgao4@lakeheadu.ca (C.G.); 2016401003@st.gxu.edu.cn (S.W.); LiuBaojie@st.gxu.edu.cn (B.L.); yaoshuangquan@gxu.edu.cn (S.Y.); 2 Guangxi Key Laboratory of Clean Pulp & Papermaking and Pollution Control, Nanning 530004, China; 3 Chemical Engineering Department, Lakehead University, Thunder Bay, ON P7B 5E1, Canada; lzhou12@lakeheadu.ca; 4 School of Chemical Engineering, Guizhou Minzu University, Guiyang 550025, China; Daiyi2020@gzmu.edu.cn

**Keywords:** chitosan, nanocrystalline cellulose, bionanocomposite film, barrier properties, mechanical properties

## Abstract

In this study, we incorporated 2,3-dialdehyde nanocrystalline cellulose (DANC) into chitosan as a reinforcing agent and manufactured biodegradable films with enhanced gas barrier properties. DANC generated via periodate oxidation of cellulose nanocrystal (CNC) was blended at various concentrations with chitosan, and bionanocomposite films were prepared via casting and characterized systematically. The results showed that DANC developed Schiff based bond with chitosan that improved its properties significantly. The addition of DANC dramatically improved the gas barrier performance of the composite film, with water vapor permeability (WVP) value decreasing from 62.94 g·mm·m^−2^·atm^−1^·day^−1^ to 27.97 g·mm·m^−2^·atm^−1^·day^−1^ and oxygen permeability (OP) value decreasing from 0.14 cm^3^·mm·m^−2^·day^−1^·atm^−1^ to 0.026 cm^3^·mm·m^−2^·day^−1^·atm^−1^. Meanwhile, the maximum decomposition temperature (Td_max_) of the film increased from 286 °C to 354 °C, and the tensile strength of the film was increased from 23.60 MPa to 41.12 MPa when incorporating 25 wt.% of DANC. In addition, the chitosan/DANC (75/25, wt/wt) films exhibited superior thermal stability, gas barrier, and mechanical strength compared to the chitosan/CNC (75/25, wt/wt) film. These results confirm that the DANC and chitosan induced films with improved gas barrier, mechanical, and thermal properties for possible use in film packaging.

## 1. Introduction

Since the 1950s, over 6.3 billion tons of plastic waste has been generated on Earth, most of which accumulate in the environment [1]. As these plastic materials are not biodegradable, their extensive production has led to the contamination of natural resources, causing serious problems, such as disturbance of the ecological balance, water pollution, and global warming [2]. In recent years, increasing attention has been paid to the development and application of biobased packaging materials derived from renewable sources that are abundant, biodegradable, inexpensive, and release low levels of carbon dioxide [3,4,5]. Biobased packaging materials can be generated from biodegradable polymers [6,7]. In this regard, significant efforts have been made to efficiently utilize cellulose and chitin, two of the most abundant polysaccharides in nature, for the production of biodegradable and non-toxic packaging materials [8,9,10].

Chitosan obtained by N-deacetylation of chitin (1,4-linked-2-amino-deoxy-β-d-glucan, IUPAC), a natural linear polysaccharide [11], has been widely used in various applications because of its safety, biocompatibility, biodegradability, flocculation ability, and excellent film-forming ability [12,13,14]. Chitosan also possesses antifungal activity and wound healing properties, which make it a potentially valuable material in biomedicine [6,15]. Chitosan films have been widely used as food packaging materials to prevent contamination and extend the shelf-life of food products. Despite these excellent properties, the application of chitosan-derived films is limited by their poor gas barrier performance and low thermal stability and mechanical strength.

Cellulose nanocrystal (CNC) can be used in biodegradable packaging materials because of its unique optical, rheological, and mechanical characteristics [16,17,18]. CNC has a high length-to-width aspect ratio (10 to 100), strong interconnected network structure, high transparency, and high mechanical strength and stiffness, which make it an ideal reinforcement agent for bionanocomposites [19,20,21]. Recently, a combination of CNC and chitosan has been investigated for the production of bionanocomposite films, and the results have indicated that the mechanical and water vapor barrier properties of chitosan films can be improved by the addition of CNC [22,23]. However, in the absence of any other treatment, CNC faces a major problem in its application as a reinforcing material, where CNC particles tend to agglomerate when mixed with chitosan in solutions [24]. Despite previous attempts [24], CNC is not well dispersed in the chitosan matrix, and the use of CNC in the chitosan film is limited [25,26].

The regioselective oxidation of CNC by periodate results in the conversion of two secondary hydroxyl groups in the C2 and C3 positions of glucose into aldehyde groups, generating 2,3-dialdehyde nanocrystalline cellulose (DANC) [27,28,29,30]. The periodate oxidation expands the application prospects of CNC, especially in combination with high molecular weight nitrogenous compounds, which can form primary (R_1_–CH=NH) or secondary (R_1_–CH=N–R_2_) aldimines and Schiff bases (R_1_–CR_3_=N–R_2_) [30]. There are many applications, such as metal ion adsorbents [31], hydrogels [32], and antimicrobial materials [33]. Several efforts have suggested that DANC can be combined with peptides to have a good antibacterial effect, which can prolong the preservation time of food [34,35]. The Schiff base reaction can occur between the reactive aldehyde groups of DANC and the amino group of chitosan, producing stable imine functionalities, thus promoting the compatibility of DANC and chitosan. Moreover, the formation of hydrogen bonds between the two polymers further facilitates their blending. For food packaging applications, gas barrier properties are critical, and fewer studies have focused on the gas barrier properties of DANC and chitosan films.

The primary aim of this study was to develop bionanocomposite films based on chitosan and DANC with improved gas barrier properties, mechanical strength, and thermal stability. To characterize the effect of DANC as a reinforcing agent, the morphological, thermal, mechanical, surface, and gas barrier properties of the films were studied in detail.

## 2. Materials and Methods

### 2.1. Materials and Chemicals

Microcrystalline cellulose (94%) was purchased from Sinopharm Chemical Reagent Co. Ltd. (Shanghai, China) and used as a raw material. Chitosan (molecule weight 100 kg/mol) with >95% deacetylation degree and sodium periodate (ACS reagent grade, ≥99.8%) were obtained from Aladdin (Zhejiang, China). The other chemicals used herein were all analytical grades and supplied by Sigma-Aldrich, Mississauga, Canada. Deionized water was used in all reactions. Dialysis membrane (molecular weight cut-off of 8000~14,000 g/mol) was obtained from Union Carbide (Texas City, TX, USA).

### 2.2. Preparation of DANC Suspension

CNC was prepared by sulfuric acid hydrolysis of microcrystalline cellulose as previously described [36]. The CNC suspension was then oxidized using sodium periodate [37]. In this research, 150 g of CNC suspension (2.50 g dry weight) was added to a 1000 mL flask and oxidized with 28 mmol of sodium periodate at 60 °C for 2 h. The flask was covered with aluminum foil to avoid light exposure. The dialdehyde nanocrystalline cellulose, DANC, was then placed into dialysis membranes, dialyzed until the conductivity of the filtrate was less than 2 μS·cm^−^^1^, and stored at 4 °C. DANC with an aldehyde content of 8.78 mmol·g^−1^ was obtained. The determination of the aldehyde group content of DANC was based on oxime reaction between the aldehyde group and hydroxylamine hydrochloride, as previously reported [38,39]. The carboxylate content of DANC was 0.014 mmol·g^−1^, as determined via a conductometric titration method, as described in the literature [40,41].

### 2.3. Preparation of Chitosan/DANC Composite Films

**Scheme 1:**
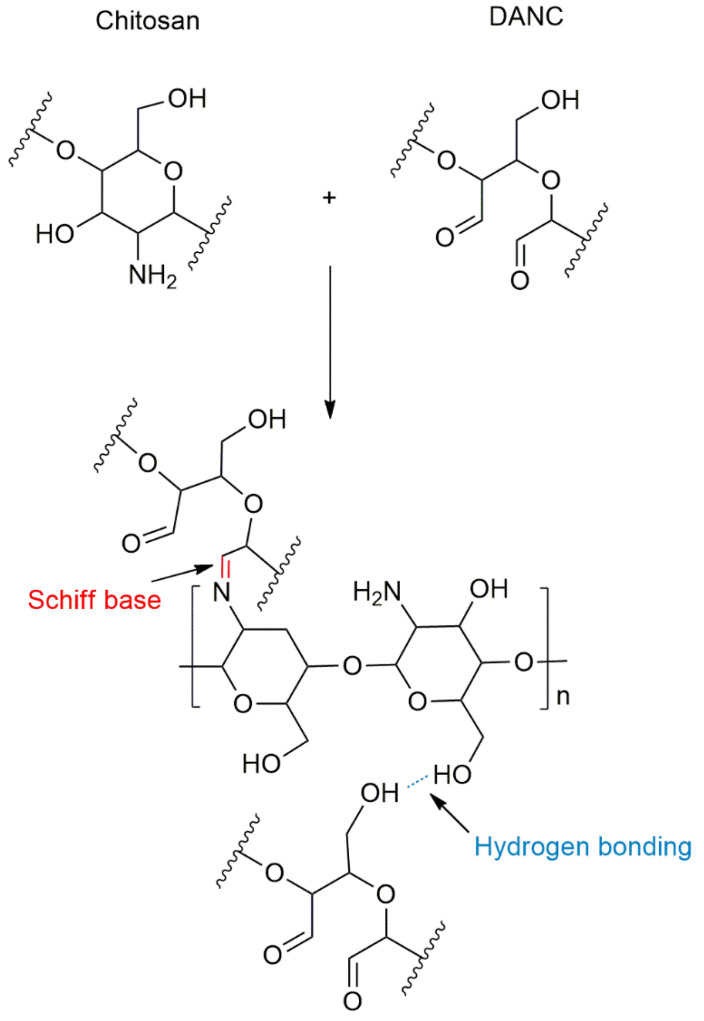
Schematic of reaction of chitosan and DANC.

Bionanocomposite films were prepared by the casting method. To obtain a 2 wt.% suspension of chitosan, chitosan powder was dissolved in a 1 wt.% acetic acid solution at 50 °C for 30 min under stirring. The solution was kept at room temperature for 24 h to stabilize the solution. The suspension was blended with various amounts of DANC (5–50 wt.%), and the mixture was stirred at 10,000 rpm for 5 min using an Ultra-Turrax mixer (IKA T10, Germany) to ensure full dispersion, degassed, and cast in an acrylic board (10 cm × 10 cm); the weight of the film was maintained at 25 g·m^−2^. The composite film was dried at 40 °C for 72 h and then stored at 20 °C and 50% relative humidity (RH). A schematic illustration of the reaction route and linking reaction between chitosan and DANC is shown in Scheme 1.

### 2.4. Characterization

#### 2.4.1. X-ray Diffraction (XRD)

The XRD analysis was recorded with a Smartlab diffractometer (Rigaku, Akishima, Japan) with a Cu Kα radiation (λ = 0.154 nm). The operating voltage and current were 40 kV and 30 mA, respectively. The diffraction angle (2θ) was collected from 5° to 40° at a scanning speed of 0.02°/s. Samples were freeze-dried before analysis. The crystallinity index (CrI) of the CNC and DANC samples were calculated using the following Equation (1) [42]:

(1) $$\mathrm{CrI}=\frac{I_{002}-I_{\mathrm{am}}}{I_{002}}$$

where I_002_ represents the intensity of the diffraction peak of the main crystal plane (I_002_, 2θ = 22.8°) and I_am_ represents the intensity of the amorphous halo (I_am_ 2θ = 18°), respectively.

#### 2.4.2. X-ray Photoelectron Spectroscopy (XPS)

The XPS analysis of the chitosan/DANC (75/25, wt/wt) film was performed using a photoelectron spectrometer (K-Alpha, Thermo fisher scientific, Waltham, USA). In this experiment, 10 mg of dried samples were used directly. Spectra were recorded using a monochromatic Al Kα X-ray source (1486.8 eV) operating at 15 kV, 10mA with a background pressure of 2 × 10^−9^ mbar. The high-energy resolution spectra were acquired using an energy analyzer operating at a resolution of approximately 0.65 eV and a pass-energy of 187 eV. The binding energy scale was calibrated by the C 1s line of aliphatic carbon, which was set at 284.8 eV. Full-spectrum, narrow high-energy resolution spectra, elemental composition, and functional groups were assessed using Avantage software (2020).

#### 2.4.3. Transmission Electron Microscopy (TEM)

The morphology of the CNC and DANC samples was obtained by TEM (HT7700, Hitachi, Tokyo, Japan) at an accelerating voltage of 100 kV. One drop of the diluted suspension (0.01 wt.%) was placed on carbon-coated copper grids and dried at room temperature before observation.

#### 2.4.4. Fourier Transform Infrared Spectroscopy (FTIR)

FTIR spectra of the resultant films were analyzed using a Tensor II spectrometer (Bruker, Berlin, Germany) in the ATR mode, with spectral width ranging from 4000 to 500 cm^−1^ at the resolution of 4 cm^−1^, and 32 scans per sample were conducted.

#### 2.4.5. Scanning Electron Microscopy (SEM)

Microstructure analysis of the surface and cross-section of the films was performed by SEM using scanning electron microscopy (F16502, Phenom Pro, Eindhoven, Netherlands). For cross-section imaging, films were quickly frozen in liquid nitrogen to make them brittle. After breaking, the samples were sputtered with gold and were examined with SEM at the accelerating voltage of 5 kV.

#### 2.4.6. Optical Transmittance Measurement

The optical transmittance of the films was measured at the wavelength range of 300–800 nm using a UV-visible spectrophotometer (Specord 50 Plus, Analytik, Jena, Germany) at the scanning speed of 50 nm·s^−^^1^.

#### 2.4.7. Thermogravimetric Analysis (TGA)

For thermogravimetric analysis, samples were weighted at approximately 10 mg in alumina cups and were heated in a pure nitrogen atmosphere (flow rate 20 mL·min^−1^) from room temperature to 800 °C at a rate of 10 °C·min^−1^ and examined in a synchronous thermal analyzer (Sta449f5, Netzsch, Gerätebau GmbH, Selb, Germany).

#### 2.4.8. Thickness and Mechanical Properties

Film thickness was examined using a digital micrometer (S16502, Frank-PTI, Birkenau, Germany) at ten random positions along with the film, and the average thickness was calculated. Tensile strength (TS) and elongation at break (Eb) tests were performed using a Lorentzen & Wettre strength tester (LS1, Ametek, Berwyn, PA, USA) with a 100 N load transducer. Films were cut into 25 mm × 80 mm strips and tested at a span length of 50 mm. The moving clamp speed was set as 0.5 mm·s^−1^. The results were calculated based on at least five measurements.

#### 2.4.9. Oxygen Permeability (OP)

The OTR of the films was determined using an oxygen permeability analyzer equipped with a coulometric oxygen sensor (GDP-C, Brugger, Feinmechanik GmbH, Munich, Germany) according to the ISO method (ISO 2556:1974). The films were exposed to 100% oxygen (purity over 99.999%) on one side and a mixture of 98% nitrogen and 2% hydrogen on the other. Oxygen permeability (OP) values (cm^3^·mm·m^−2^·day^−1^·atm^−1^) were determined by multiplying the OTR (cm^3^·m^−2^·day^−1^) value by the film thickness and dividing it by the partial pressure difference of oxygen (1 atm) [11,43]. The specimen area was 78.4 cm^2^, and measurements were performed in duplicate at 23 °C.

#### 2.4.10. Water Vapor Permeability (WVP)

The water vapor transmission rate (WVTR) was measured in a water vapor permeability tester (TSY-T1, TOYOC, Dongguan, China) using a standard method (ASTM E96). Before testing, films were incubated for 24 h at 20 °C and 50% RH (as saturated water vapor transmits through films at a certain temperature and humidity); the films were then cut into discs with a diameter of 100 mm, and the WVTR was determined as a decrease in distilled water weight with time (g·m^−2^·day^−1^) according to the formula:

WVTR = Δm/(A × t)(2)

where Δm is distilled water weight difference, t is the time between two weight measurements, and A is the exposed film area [44,45].

The WVP of the film was calculated by multiplying the WVTR value by the film thickness and dividing that by the water vapor pressure difference across the film. The unit of WVP is given in g·mm·m^−2^·atm^−1^·day^−1^ [46].

## 3. Results and Discussion

### 3.1. Characterization of CNC and DANC

The aldehyde and carboxylate contents of CNC and DANC samples are provided in Table 1. Due to the periodate oxidation, 8.78 mmol/g of aldehyde groups were introduced into DANC. Conversely, after periodate oxidation, the carboxylate content of the DANC sample increased from 0.0065 to 0.014 mmol/g. The yield of the CNC preparation process was 67.2% and the yield of the DANC reaction was 79.2%.

**Figure 1:**
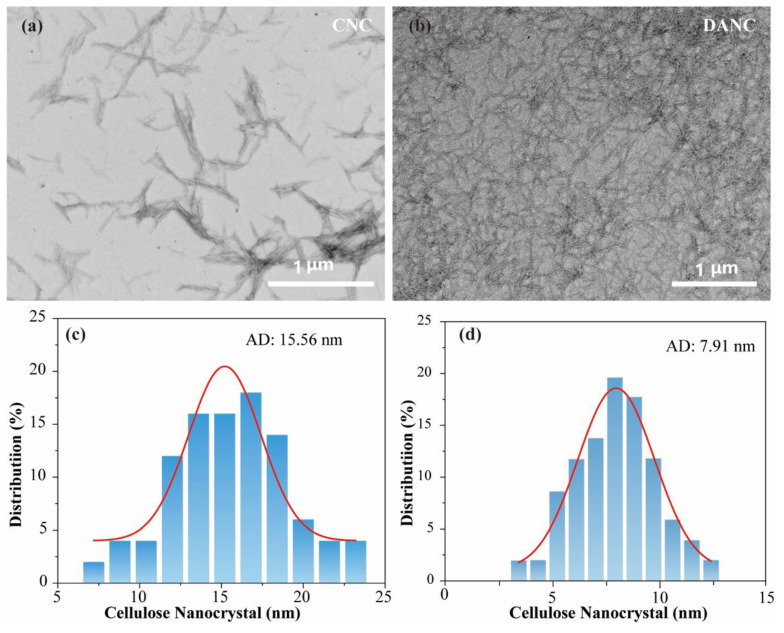
The TEM images of CNC and DANC: (**a**) CNC, (**b**) DANC, and the particle size distribution of (**c**) CNC and (**d**) DANC.

The morphology and size of CNC and DANC were investigated by TEM, and images are exhibited in Figure 1. No aggregation was found between nanoparticles, and both CNC and DANC exhibited a rod-like morphology with a large aspect ratio. CNC particles had a length of approximately 200~500 nm and a diameter of approximately 15.56 nm (Figure 1c). DANC particles were found to be more uniform, with a smaller size. The diameter of DANC particles was approximately 7.91 nm (Figure 1d). Similar results were reported in the literature [47,48].

**Figure 2:**
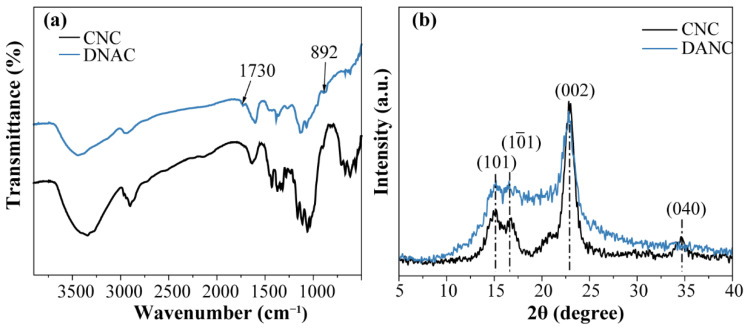
(**a**) FTIR spectra of CNC and DANC, (**b**) XRD spectra of CNC and DANC.

The FTIR spectra of CNC and DANC are shown in Figure 2a. The positions of characteristic peaks in the CNC and DANC spectrum were similar to those observed in previous studies [39,49]. The appearance of two new absorption bands proved that aldehyde groups were successfully grafted onto DANC; the band of 1730 cm^−1^ and 892 cm^−1^ attributed to the C=O stretching vibration absorption peak and the hemiacetal bond, respectively [30,39]. In addition, the characteristic absorption peak at 1060 cm^−1^ is the stretching vibration of the primary hydroxyl group, and the intensity of the corresponding absorption peak in DANC is relatively weak, which would indirectly indicate the oxidation between the CNC molecules. It is noteworthy that the stretching vibration of O–H at 3347 cm^−1^ and aliphatic C–H at 2920 cm^−1^ of DANC is slightly broader but weaker than those on the CNC spectrum. It has been reported [50] that the hydrogen bonds are arranged axially in the cellulose type I structure, and the periodate oxidation would disrupt the original hydrogen bonding distribution and create many new disordered hydrogen bonds, which would lead to the broadening of the stretching vibration of O–H.

The X-ray diffraction patterns of CNC and DANC are presented in Figure 2b. The two samples correspond to typical cellulose type I structures with major 2θ diffraction angles close to 15.1°, 16.6°, and 22.5°; these peaks correspond to 101, 1$\bar{0}$1, and 002 crystalline planes, respectively. The crystal structure of DANC did not rearrange after oxidation, which is in agreement with that reported by Varma et al. [51]. The CrI of CNC was 94.40% (Table 1), which indicated that it had high crystallinity. After the periodate oxidation, the main peak of DANC changed into a broad diffraction peak and the CrI of DANC decreased to 86.02%. This can be attributed to the fact that periodate oxidation would result in the ring-opening of the glucopyranose and thus the disruption of the order structure of cellulose molecules [52,53].

### 3.2. FTIR Analysis of DANC Containing Film

**Figure 3:**
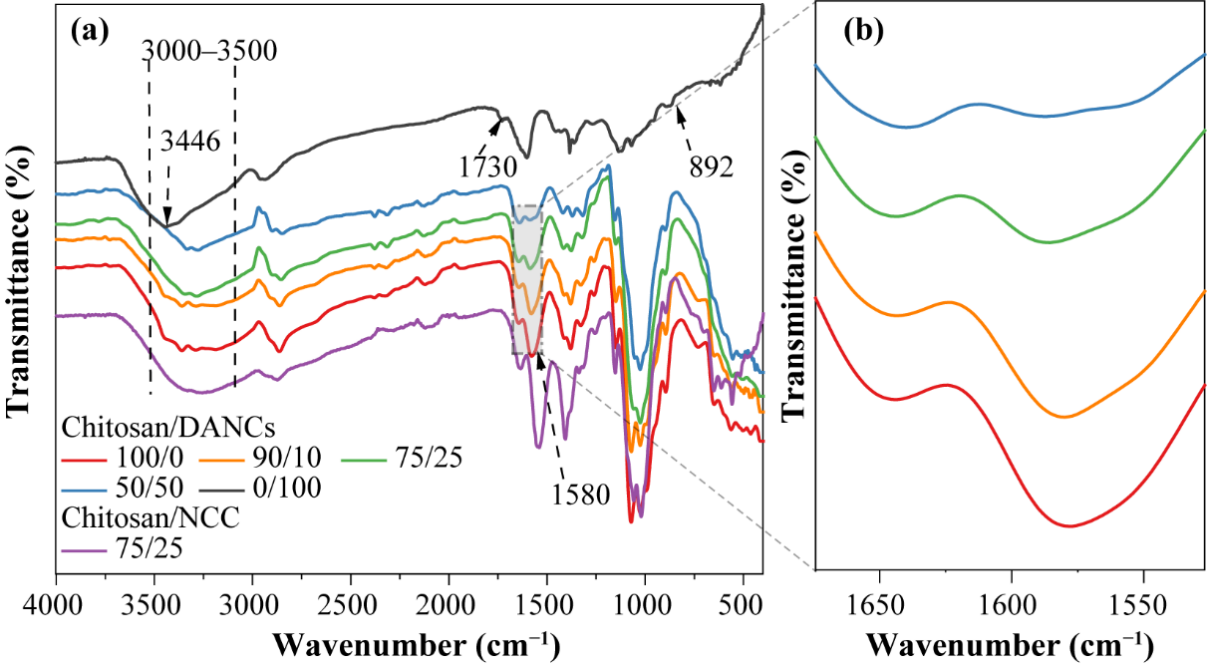
FTIR spectra of chitosan/DANC and chitosan/CNC films, (**a**) wavenumber range of 450 to 4000 cm^−1^, (**b**) magnified wavenumber of 1500–1700 cm^−1^. Numbers indicate relative content (wt/wt) of chitosan, DANC, and CNC in the films, respectively.

FTIR analysis was performed to understand the interaction of DANC and chitosan and assess the effect of DANC incorporation into the chitosan films. Figure 3a shows the FTIR spectra of chitosan/DANC films with different DANC contents, while Figure 3b shows the magnified spectra of the same samples in the range of 1700-1500 cm^-1^. The characteristic absorption peaks of chitosan at 1644 cm^−1^ (C=O stretching in amide I), 1580 cm^−1^ (–NH_2_ bending), and 1380.4 cm^−1^ (–CH_2_ bending) were observed [54]. The peaks that appeared at 1150 cm^−1^ (anti-symmetric stretching of the C-O-C bridge) and 1073 cm^−1^ (skeletal vibrations involving the C–O stretching) correspond to the saccharide structure of chitosan [55,56]. The positions of characteristic peaks in the chitosan spectrum were similar to those observed in previous studies [57,58,59]. A broad absorption peak from 3000 to 3500 cm^−1^ was mainly associated with the O–H stretching vibration and overlapped with –NH_2_ stretching, whereas a peak at around 2860 cm^−1^ was attributed to aliphatic C–H stretching vibration [60]. Two characteristic absorption peaks at 1730 cm^−1^ and 892 cm^−1^ were observed in the spectrum of DANC films [27,39]. The first one was attributed to C=O generated by the periodate oxidation of CNC. The other one was because the periodate oxidation process led to the formation of hemiacetal bonds between the aldehyde groups and neighboring –OH [30,45]. The FTIR spectrum of the pure DANC film revealed absorption peaks at 3440 cm^−1^ and 2920 cm^−1^, which were related to O–H and aliphatic C–H stretching vibrations, respectively.

Compared with the chitosan/CNC film, the addition of DANC caused changes in the FTIR spectra of the chitosan/DANC films. The periodate oxidation resulted in the conversion of two hydroxyl groups in the C2 and C3 positions of glucose into aldehyde groups [30]. A Schiff base bond was formed between the reactive aldehyde groups of DANC and amino groups of chitosan. As DANC content was increased, more aldehyde groups were bound to the amino group of chitosan. Because of the Schiff base reaction, the peak of DANC at 1730 cm^−1^ (aldehydic carbonyl groups) and 890 cm^−1^ (hemiacetal bond) vanished after introducing DANC into chitosan. As the content of DANC increased, the intensity of the –NH_2_ stretching peak at 3450 cm^−1^ decreased gradually. When the amount of DANC reached 50%, the peak at 3448 cm^−1^ disappeared. Concurrently, there was a dramatic decrease in the intensity of the absorption band of –NH_2_ bending at 1580 cm^−1^ as the content of DANC increased. The peak of –C=N stretching vibration at 1640 cm^−1^ formed by the Schiff base reaction overlapped with the C=O stretching in the amide I of chitosan. Overall, our results indicated that DANC was successfully introduced into the chitosan matrix. As DANC and chitosan have similar molecular structures, it is expected that the two polymers should have good miscibility and compatibility [61].

### 3.3. XPS Analysis of DANC Containing Film

**Figure 4:**
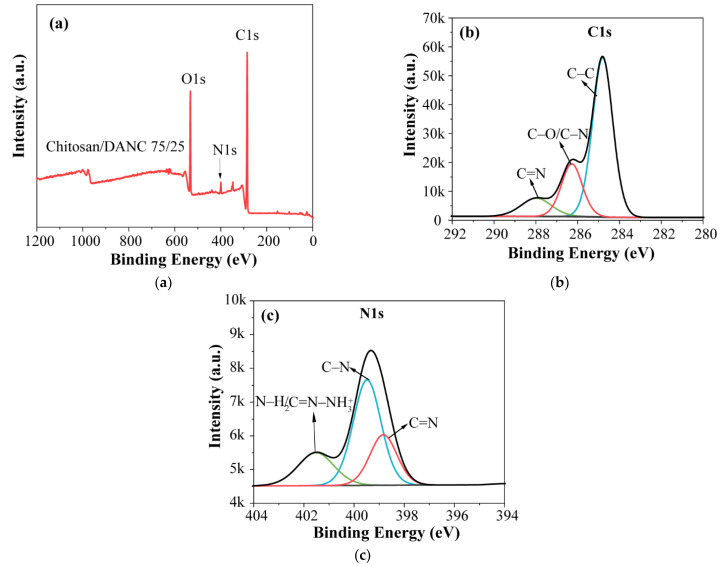
XPS spectra of chitosan/DANC (75/25, wt/wt) film: (**a**) full scans, (**b**) C1s scans, and (**c**) N1s scans.

To further investigate the surface composition and covalent linkage of the composite films, X-ray photoelectron spectroscopy (XPS) of the films was studied. As shown in Figure 4a, the chitosan/DANC (75/25, wt/wt) film showed two major peaks of C 1s (285.2 eV) and O 1s (532.3 eV) as well as a minor peak that can be assigned to N 1s (399.5 eV) signals. The high-resolution XPS spectra of C 1s with the fitting data are presented in Figure 4b. The C 1s spectrum can be deconvoluted into three sub-peaks. The main peak located at 284.8 eV was attributed to the C–C bond [62]. The second peak can be observed at 286.3 eV, which indicated the presence of C–N and C–O bonds [63]. The third peak at 288.8 eV was attributed to C=N and O=C–O bonds [64,65]. The high-resolution N 1s spectrum (Figure 4c) was composed of three peaks. The peak at 399.5 eV was attributed to the C–N bond. The shoulder peak at 398.8 eV was assigned to the binding energy of the C=N bond [64,66], suggesting that a Schiff base reaction (Scheme 1) of amino groups and aldehyde groups occurred, which was consistent with the FTIR results (Figure 3). It should be noted that a minor peak was observed at 401.5 eV, which could be either an N–H_2_ bond in the chitosan, as shown in Scheme 1, or a protonated amine nitrogen (C=${N–\mathrm{NH}}_{3}^{+}$) [64,67]. Based on the results, DANC was successfully incorporated into the chitosan matrix via the Schiff base reaction.

### 3.4. Thermal Gravimetric Analysis of DANC Containing Film

**Figure 5:**
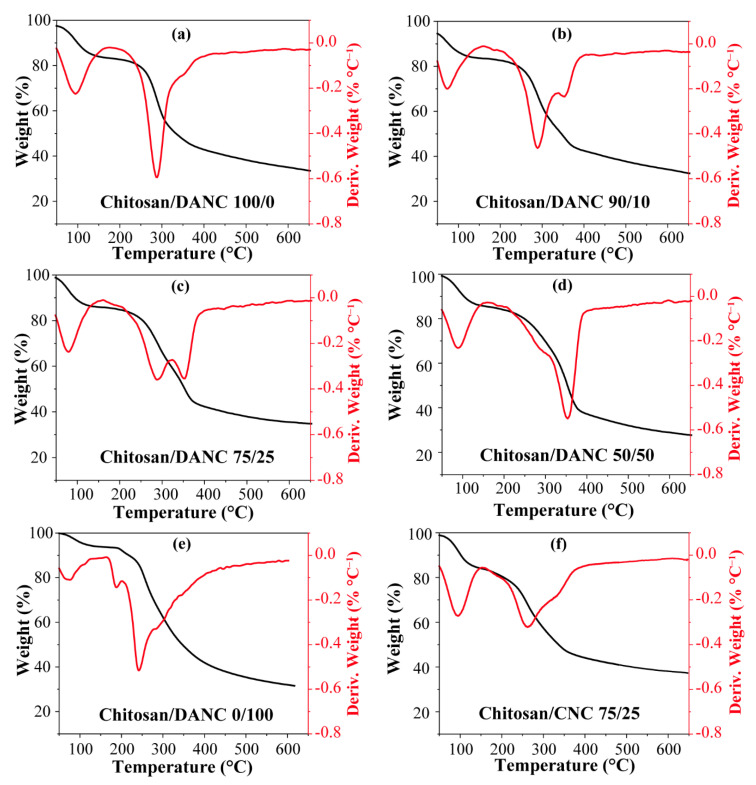
Thermal gravimetric (TG) and derivative thermal gravimetric (DTG) curves for chitosan, chitosan/DANC, and chitosan/CNC films: (**a**) chitosan/DANC 100/0, (**b**) chitosan/DANC 90/10, (**c**) chitosan/DNAC 75/25, (**d**) chitosan/DANC 50/50, (**e**) chitosan/DANC 0/100, and (**f**) chitosan/CNC 75/25. Numbers indicate relative content (wt/wt) of chitosan, DANC, and CNC in the films, respectively.

The thermal stability of the films was analyzed, and the results are shown in Figure 5a–f. The results of TGA revealed two major weight losses. The initial drop in the temperature range of 50~200 °C was attributed to the evaporation of adsorbed water and acetic acid. Weight losses for chitosan (100/0) and chitosan/DANC (90/10, 75/25 and 50/50, wt/wt) films were 17.31%, 17.41%, 15.26%, and 16.10%, respectively. The second thermal event resulting in a major weight loss in all films was observed in the temperature range of 200~400 °C (Figure 5a), and this trend could be attributed to the fast volatilization of polymer segments due to the thermal scission of the polymer backbone [68]. In an inert atmosphere, there was only one main peak in the thermal degradation curves of pure chitosan film and DANC film, while two main peaks were observed in the curve of chitosan/DANC (90/10, 75/25, 50/50 wt/wt) films. The maximum decomposition temperature (Tdmax) of these steps was determined from the DTG results. The Tdmax for pure chitosan film was 286 °C, while the Tdmax of chitosan/DANC (50/50 wt/wt) film was 354 °C. The Tdmax of chitosan/CNC film was 266 °C (Figure 5f), and the CNC addition insignificantly affected the thermal behavior of the chitosan films [6,11]. In the chitosan/DANC (90/10, 75/25, wt/wt) films (Figure 5b,c), two degradation steps were observed with Tdmax at 286 and 354 °C. By increasing the DANC content in the chitosan film, the Tdmax of the chitosan/DANC films shifted to higher temperatures, indicating that DANC improved the thermal stability of the film. In this case, the presence of the crystalline structure and great compatibility of chitosan and DANC improved the thermal stability of the chitosan/DANC films. In addition, the Schiff base reactions that occurred between DANC and chitosan significantly improved the thermal stability of the composite film. Compared to the pure chitosan film with the maximum weight loss rate of 0.59%·°C^−1^, the chitosan/DANC (75/25, wt/wt) had a 0.35%·°C^−1^ weight loss rate. Similar results have been reported in previous studies [69]. In all films, the weight loss tended to become stable gradually at the temperature range of 400~600 °C. At 600 °C, the residual amounts of the films with 100/0, 90/10, 75/25, 50/50, and 75/25 (wt/wt) chitosan/CNC film content were 35.06%, 34.21%, 35.53%, 28.9%, and 38.08%, respectively. Generally, the better degradation performance of chitosan/DANC films was related to the formation of strong hydrogen bonds between the –OH groups of DANC and the free –OH groups of chitosan and the Schiff reaction (Scheme 1) [70,71].

### 3.5. Optical Properties of the DANC Containing Films

**Figure 6:**
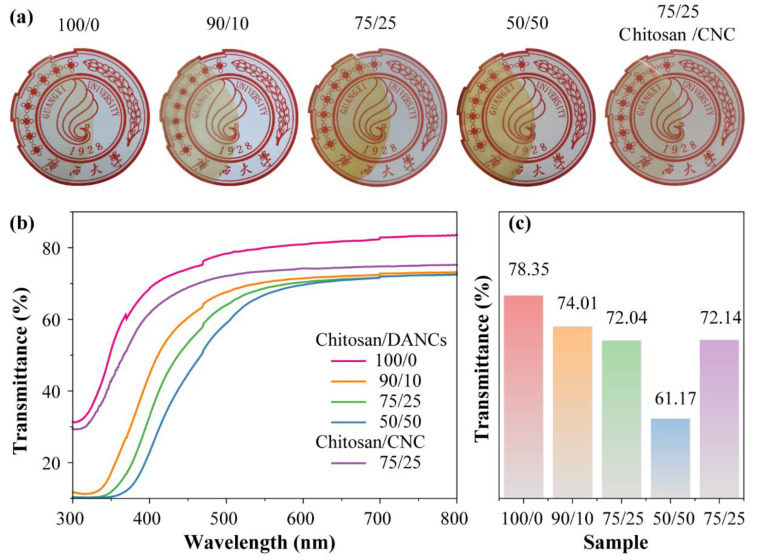
(**a**) Photograph of composite films; (**b**) Optical transmittance of composite films with different chitosan, chitosan/DANC, and chitosan/CNC films; (**c**) Optical transmittance of composite films at 500 nm.

Transparency and gloss are important characteristics of films. The optical properties of films are directly related to homogeneity and internal microstructure of the matrix and distribution of DANC in the films [72]. The photographs and optical transmittance curves of chitosan, chitosan/DANC, and chitosan/CNC films are shown in Figure 6a. As discussed, the higher the chitosan content, the higher the transmittance of the film [11,73]. The printed letters “Guangxi” are visible for all sample sets. All composite films have a yellowish appearance. As the DANC content increased, the word “Guangxi” became unclear. The UV-Vis analysis indicated that all chitosan films exhibited very high transparency, with transmittance over 60% in the visible light range (380~740 nm) (Figure 6b). The chitosan/DANC films exhibited better light transmission than reported in previous studies, where transparency was reduced to under 40% at 50 wt.% cellulose nanofiber inclusion in the chitosan films [74,75]. The addition of DANC to composite films gradually decreased transparency, especially in the range below 600 nm (Figure 6b). The pure chitosan (100/0) film presented the highest optical transparency of 78.35% at 500 nm, whereas in the 90/10, 75/25, 50/50, and 75/25 chitosan/DANC film, it was 74.01%, 72.04%, 61.17%, and 72.14%, respectively (Figure 6c). This behavior could be related to the slight aggregation of DANC particles in the chitosan matrix and the formation of a Schiff base structure between chitosan and DANC, which would increase the structural density of the film and thus scatter the light more intensely than the pure chitosan and chitosan/CNC films do [76].

### 3.6. Mechanical Properties

**Figure 7:**
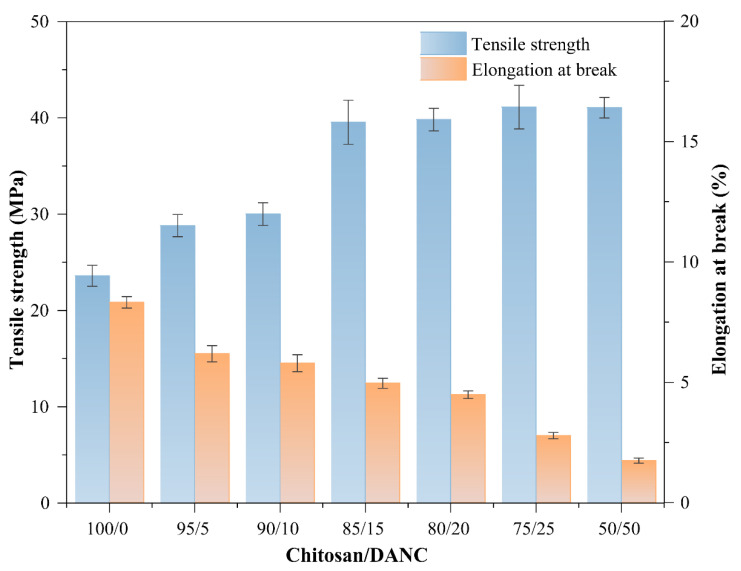
Effect of DANC content on the TS and Eb parameters of chitosan/DANC films. Numbers indicate relative content (wt/wt) of chitosan and DANC in the films, respectively.

The tensile strength (TS) and elongation at break (Eb) values of chitosan/DANC films are presented in Figure 7, which shows that the TS was increased by the addition of DANC. In other words, the TS, which was 23.60 MPa for the pure chitosan, was increased by the addition of 5, 10, 15, 20, 25, and 50 wt.% DANC to 28.80, 30.02, 39.54, 36.82, 41.12, and 41.06 MPa, respectively. The improvement in the tensile strength of the composite film can be attributed to the addition of DANC. The highest TS value obtained was 41.1 MPa with 25 wt.% DANC addition, which was much higher than the 14.2 MPa obtained with the chitosan/CNC (75/25 wt/wt) film (Table 2). Similar results were reported for the other films containing cellulose derivatives. For example, by incorporating 20 wt.% oxidized cellulose nanofibers into chitosan film, the tensile stress was changed from 10.7 to 18.7 MPa [11]. The increase in the TS for chitosan/DANC films can be attributed to two factors: (1) a large number of active hydroxyl groups in DANC and chitosan molecules, as shown in Scheme 1 [72]; (2) the Schiff base reactions between the aldehyde group of DANC and the amine group of chitosan [24,44,77]. In addition, the reinforcing effect due to efficient stress transfer at the chitosan/DANC interface might lead to the strengthening of the composite film [78]. Such interactions between nanocomposite film components could restrict the segmental mobility of the polymer chains in the vicinity of the nano reinforcement [11,79]. The results indicate that DANC can be used for the reinforcement and improvement of the mechanical strength of chitosan-based films, as the formation of good mechanical cohesion is a prerequisite for the development of new composite materials.

The Eb value was found to be 8.32% for the pure chitosan film and 6.19%, 5.79%, 4.96%, 4.48%, 2.79%, and 1.75% for 95/5, 90/10, 85/15, 80/20, 75/25, and 50/50 (wt/wt) chitosan/DANC films, respectively, indicating that the Eb in films steadily decreased with the addition of DANC to chitosan. The addition of DANC had a negative effect on the brittleness of the composite films. In other studies of chitosan films, a general trend of decreasing Eb value had been observed when the reinforcing material was a nanocellulose based material [6,11,77]. The decrease in Eb value can be affected by the volume fraction of the added reinforcing material, the dispersion in the matrix, and the strong interactions between chitosan and DANC restricting the motion of the matrix [23,80,81]. A similar phenomenon was observed in the study of Wang et al. [3]. The addition of DANC has significantly increased the tensile strength of the composite film without significantly affecting the flexibility of the composite film.

### 3.7. Gas Barrier Properties

**Figure 8:**
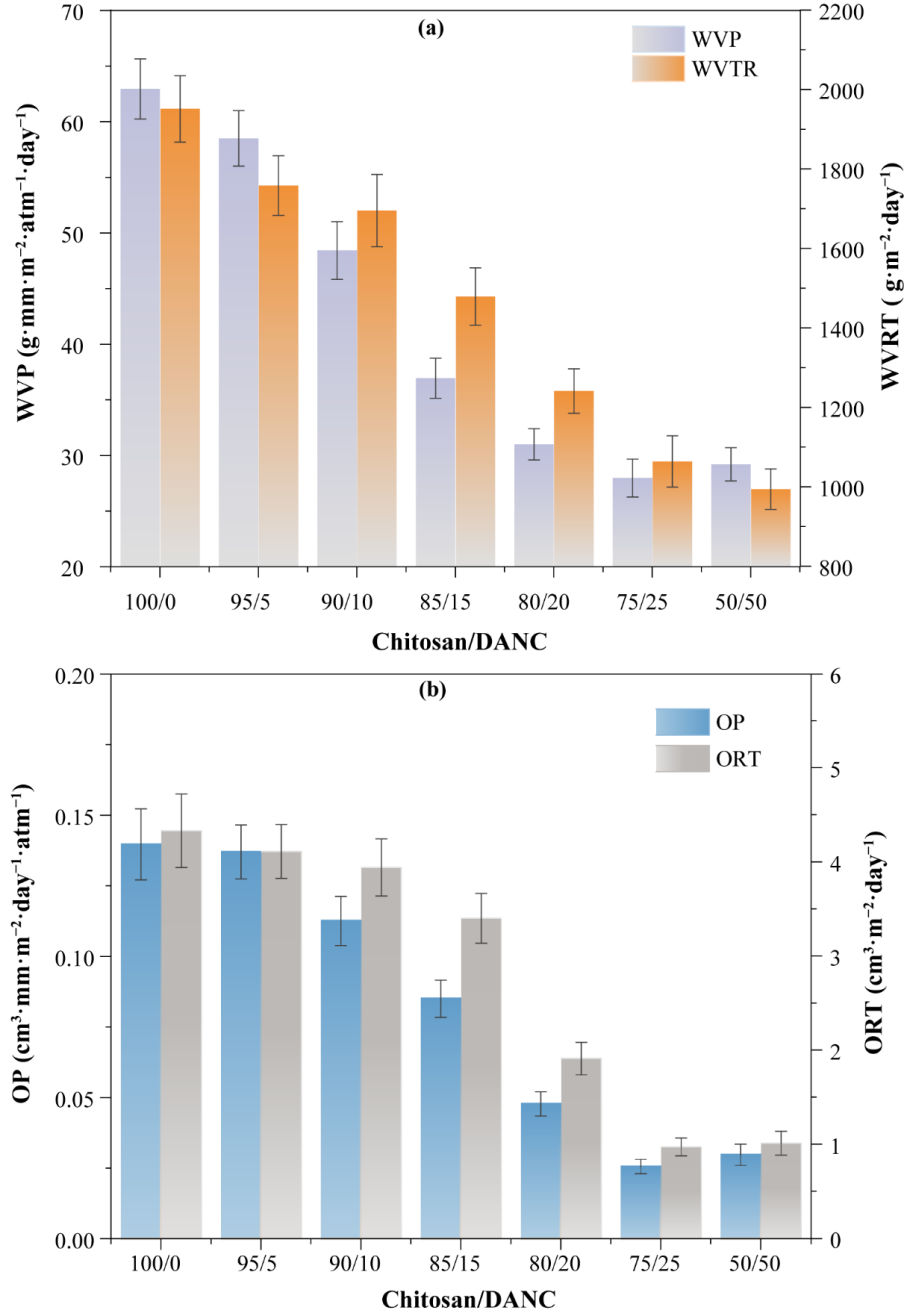
Effect of DANC content on the (**a**) WVP, WVRT, and (**b**) OP, ORT value of the chitosan/DANC films. Numbers indicate relative content (wt/wt) of chitosan and DANC in the films, respectively.

The WVP is one of the most important features of films for their application in the food packaging industry. The WVP is influenced by a diffusion process in which water vapor first condenses and dissolves on the film surface and then droplet water diffuses through the film [82]. Moisture transfer between food and the surrounding atmosphere must be prevented by the films, which is essential for their application in food packaging. The results of the WVP testing in the composite films indicated that DANC caused a reduction in the WVP of the chitosan-based films (Figure 8). The WVP of the pure chitosan film (62.94 g·mm·m^−2^·atm^−1^·day^−1^) was slightly decreased by the addition of 5% DANC (to 58.51 g·mm·m^−2^·atm^−1^·day^−1^). This may be attributed to the fact that the DANC concentration was too low to form a sufficient amount of hydrogen bonds with the hydrophilic -OH groups of chitosan to form an effective shield against water. However, a further increase in the DANC content resulted in a dramatic decrease in the WVP (Figure 8a). In this case, the WVP was reduced from 58.51 g·mm·m^−2^·atm^−1^·day^−1^ in the 95/5 wt/wt film to 27.97 g·mm·m^−2^·atm^−1^·day^−1^ in the 75/25 wt/wt film. This phenomenon can be attributed to the fact that increasing the incorporation of DANC into the chitosan matrix strengthened the interaction between the two polymers, which slowed moisture diffusion and reduced permeability. It is known that moisture diffuses more easily through amorphous areas of the polymer matrix; therefore, highly crystalline DANC well disperses in the chitosan matrix and probably blocks the moisture transmission [11,16]. In addition, a stable crosslinking system is formed between DANC and the chitosan matrix (Scheme 1), which extends the diffusion path of water molecules within the composite membrane and improves the water vapor barrier properties of the film [83]. The WVTR value was 1063.8 g·m^−2^·day^−1^ with 25 wt.% DANC addition (Figure 8a), which was 28% lower than the 1476.8 g·m^−2^·day^−1^ obtained with the chitosan/CNC (75/25 wt/wt) film (Table 2). The composite films studied in this work show similar results reported for other packaging applications [82,84,85]. It was previously reported that films with WVTR in the range of 904–1447 g·m^−2^·day^−1^ were found to be good candidates for the application of moderately exuding wounds [85]. As shown in Figure 8a, the WVRT of chitosan/CNC (80/20, 75/25) films were within the desired range, indicating that chitosan/DANC composite films also have a great potential for application in dressing materials.

Reduction of the OP, which reflects the amount of oxygen passing through a material in a given period, is crucial for food packaging, as oxygen transmission could lead to product oxidation, which would affect food quality parameters such as odor, color, flavor, and nutrient content [86]. Nanocomposite films have been shown to possess excellent oxygen barrier properties, which can help improve food quality and extend food shelf-life [30,87,88]. The OP of chitosan-based composite films with various DANC contents demonstrated that oxygen transmission in composite films was significantly reduced with the addition of DANC (Figure 8b). Thus, the pure chitosan film had an OP of 0.14 cm^3^·mm·m^−2^·day^−1^·atm^−1^, whereas the addition of 5, 10, 15, 20, and 25% DANC decreased the OP of the films to 0.13, 0.11, 0.085, 0.048, and 0.025 cm^3^·mm·m^−2^·day^−1^·atm^−1^, respectively. A possible reason for such an OP reduction by DANC may be the strong hydrogen bonding between DANC and chitosan molecules, which would lead to the formation of a compact structure of the films, preventing gas permeation [76]. Furthermore, a decrease in the free volume or densification of the network in the polymeric matrix would generate a tortuous path for oxygen, which would consequently decrease permeability [89]. The OTR value of chitosan/DANC films is shown in Figure 8b. Generally, when the OTR of the film was less than 10 cm^3^·m^−2^·day^−1^, it could be regarded as a material with an acceptable oxygen barrier property [11,90]. All composite films in this study met this standard. The OTR for the chitosan/DANC (75/25, wt/wt) film was 0.97 cm^3^·m^−2^·day^−1^, which was less than that of the pure chitosan film (4.33 cm^3^·m^−2^·day^−1^) and the chitosan/CNC (75/25, wt/wt) (3.29 cm^3^·m^−2^·day^−1^) film (Table 2). Based on the oxygen barrier properties of other common commercial packaging film materials, such as polyethylene terephthalate (PET) (110 cm^3^·m^−2^·day^−1^), polyamideethylene vinyl alcohol (PA-EVOH-PA) (0.5 cm^3^·m^−2^·day^−1^), and polypropylene-ethylene vinyl alcohol (PP-EVOH-PP) (0.3 cm^3^·m^−2^·day^−1^), the oxygen barrier performance of chitosan/DANC films are acceptable [11]. Overall, these results indicate that chitosan/DANC nanocomposite films could be used as a biocompatible food packaging material to protect food from oxidation-induced spoilage.

### 3.8. Morphology of Films

**Figure 9:**
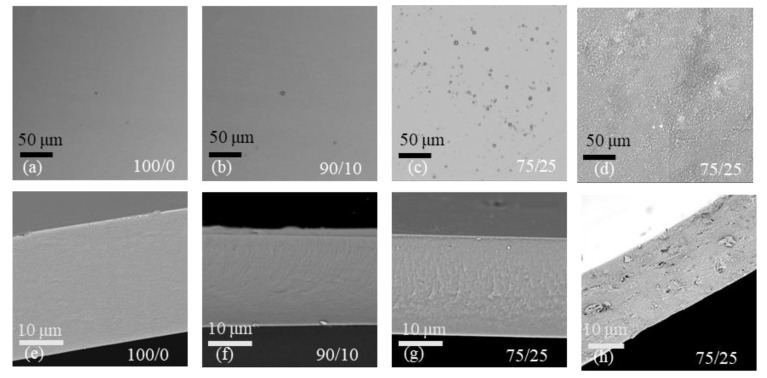
SEM images of chitosan and chitosan/DANC films. (**a**–**d**) Surface morphology of pure chitosan (**a**), chitosan/DANC films (**b**,**c**), and chitosan/CNC (**d**). (**e**–**h**) Cross sections of pure chitosan (**e**), chitosan/DANC (**f**,**g**) films, and chitosan/CNC (**h**). Numbers indicate relative content (w/w) of chitosan, DANC, and CNC in the films, respectively.

Based on the experimental results of mechanical properties, gas barrier properties, and light transmission, the dosage of 25 wt.% was selected as the optimum dosage for both DCNC and CNC. SEM can provide information regarding the distribution of aggregates, the presence of voids, the possible orientation of nanoparticles, and the degree of nanoparticle dispersion within the film [68]. Figure 9 shows the SEM images of surfaces (Figure 9a–d) and cross-sections of a pure chitosan film (Figure 9e–h), a chitosan/DANC film, and a chitosan/CNC film. All chitosan/DANC films had a homogeneous structure without macroscopic pores or cracks, indicating uniformity of the material. The addition of DANC changed the film microstructure (Figure 9b,c,f,g). The images show that DANC was evenly dispersed within the chitosan matrix, and no obvious DANC aggregation was evident, suggesting good compatibility between the two polymers in the matrix [54]. Compared to the pure chitosan film, white dots appeared on the surface of chitosan/DANC films with a DANC content of 10% (Figure 9b) and 25% (Figure 9c). This was probably due to the transversal sectioning of cellulose crystals [91]. The chitosan/CNC film (Figure 9d) showed an inhomogeneous surface with many irregular protrusions when comparing the chitosan/CNC films to the chitosan/DANC films. Cross-sectional images indicated that the pure chitosan film was smooth and compact (Figure 9e), whereas the incorporation of 25% DANC resulted in some irregularities, such as multiple ripples and ridges on the surface of the film (Figure 9g). In the cross-sectional image of all samples, the chitosan/CNC film (Figure 9h) was the roughest, and not sufficiently tight in structure. Under these observations, the good distribution and compatibility between DANC and chitosan are prerequisites for improving the mechanical properties of the films.

## 4. Conclusions

In this study, DANC was prepared through the periodate oxidation of CNC. DANC was mixed with chitosan at different concentrations as a reinforcing agent, and biocomposite films were prepared by a simple solution-casting method. The FTIR and XPS analyses confirmed that a Schiff base reaction took place between the highly reactive aldehyde group of DANC and the active amino group of chitosan and that DANC was successfully introduced into the chitosan matrix. The results showed that the Tdmax increased from 286 °C to 354 °C, and the tensile strength was increased from 23.60 MPa to 41.12 MPa when the incorporation of DANC was increased to 25 wt.% in the chitosan film. Meanwhile, the addition of DANC dramatically improved the gas barrier performance of the composite film, with the WVP value decreasing from 62.94 g·mm·m^−2^·atm^−1^·day^−1^ to 27.97 g·mm·m^−2^·atm^−1^·day^−1^ and OP value decreasing from 0.14 cm^3^·mm·m^−2^·day^−1^·atm^−1^ to 0.026 cm^3^·mm·m^−2^·day^−1^·atm^−1^ when 25 wt.% DANC was added to the chitosan film. Thus, DANC acted as an excellent reinforcing agent, and it improved the thermal stability and mechanical characteristics of chitosan/DANC composite films, which significantly enhanced the oxygen and water vapor barrier properties of the film. The findings of this study indicated that the incorporation of DANC into chitosan is a feasible and effective approach to producing biodegradable films for food packaging.

## Figures and Tables

**Table 1:** Properties of CNC and DANC.

Sample	CNC	DANC
Aldehyde content, mmol/g	–	8.78
Carboxylate content, mmol/g	0.0065	0.014
CrI, %	94.40	86.02
yield, %	67.2	79.2

**Table 2:** The tensile strength (TS), elongation at break (Eb), WVP, WVRT, OP, and ORT values of chitosan/CNC (75/25, w/w) film.

Sample	TS, MPa	Eb, %	WVP, g·mm·m^−2^·atm^−1^·day^−1^	WVRT, g·m^−2^·day^−1^	OP, cm^3^·mm·m^−2^·day^−1^·atm^−1^	ORT, cm^3^·m^−2^·day^−1^
75/25	14.2	3.05	47.64	1476.84	0.11	3.29

## Data Availability

The data presented in this study are available on request from the corresponding author.
